# White matter T2 hyperintensities and blood‐brain barrier disruption in the hyperacute stage of subarachnoid hemorrhage in male mice: The role of lipocalin‐2

**DOI:** 10.1111/cns.13221

**Published:** 2019-09-30

**Authors:** Yasunori Toyota, Jialiang Wei, Guohua Xi, Richard F. Keep, Ya Hua

**Affiliations:** ^1^ Department of Neurosurgery University of Michigan Ann Arbor MI USA

**Keywords:** blood‐brain barrier, knockout mice, lipocalin‐2, subarachnoid hemorrhage, T2 hyperintensity, white matter injury

## Abstract

**Aims:**

The current study examined whether white matter injury occurs in the hyperacute (4 hours) phase after subarachnoid hemorrhage (SAH) and the potential role of blood‐brain barrier (BBB) disruption and an acute phase protein, lipocalin 2 (LCN2), in that injury.

**Methods:**

Subarachnoid hemorrhage was induced by endovascular perforation in adult mice. First, wild‐type (WT) mice underwent MRI 4 hours after SAH to detect white matter T2 hyperintensities. Second, changes in LCN2 expression and BBB disruption associated with the MRI findings were examined. Third, SAH‐induced white matter injury at 4 hours was compared in WT and LCN2 knockout (LCN2 KO) mice.

**Results:**

At 4 hours, most animals had uni‐ or bilateral white matter T2 hyperintensities after SAH in WT mice that were associated with BBB disruption and LCN2 upregulation. However, some disruption and LCN2 upregulation was also found in mice with no T2‐hyperintensity lesion. In contrast, there were no white matter T2 hyperintensities in LCN2 KO mice after SAH. LCN2 deficiency also attenuated BBB disruption, myelin damage, and oligodendrocyte loss.

**Conclusions:**

Subarachnoid hemorrhage causes very early BBB disruption and LCN2 expression in white matter that is associated with and may precede T2 hyperintensities. LCN2 deletion attenuates MRI changes and pathological changes in white matter after SAH.

## 1 INTRODUCTION

Subarachnoid hemorrhage (SAH) is a cerebrovascular disease with particularly high mortality and morbidity rates. SAH induces rapidly elevated intracranial pressure and decreased cerebral blood flow.^1^, ^2^ SAH causes early brain injury, including oxidative stress, blood‐brain barrier (BBB) disruption and brain edema,^3^ and such edema is an important risk factor for poor clinical prognosis after SAH. In addition, we have found that experimental SAH induced by endovascular perforation induces white matter damage that can be detected by magnetic resonance imaging (MRI) as T2‐hyperintensities 24 hours post ictus.^4^ Although many studies have reported early brain injury after SAH, the exact mechanisms have not been fully elucidated.^5^, ^6^, ^7^ Studies examining very early changes (hyperacute) may help elucidate the relationship between different forms of injury. For example, ultra‐early BBB disruption was recently reported in cerebral ischemia and specifically targeting that endothelial injury could block later brain injury and long‐term behavioral deficits.^6^

One protein that might be involved in early brain injury after SAH is lipocalin‐2 (LCN2). We have previously reported a role of LCN2 in white matter injury at 24 hours after SAH.^4^, ^8^ However, LCN2 is best described as an acute phase siderophore‐binding protein that also has a role of chemokine inducer. LCN2 has been implicated in the apoptotic cell death, regulation of cell differentiation and endogenous iron chelation. Whether it is upregulated in the hyperacute phase after SAH and has a role in BBB and parenchymal injury during that phase is unknown.

The present study investigated white matter parenchymal and BBB injury in the hyperacute (4 hours) phase after SAH. It also examined the role of LCN2 by comparing SAH‐induced injury in wild‐type (WT) and LCN2 knockout mice undergoing endovascular perforation.

## 2 MATERIALS AND METHODS

### 2.1 Animal preparation

All animal protocols were approved by the University of Michigan Committee on the Use and Care of Animals. Animals were housed under standard 12:12 light‐dark conditions and allowed free water and food. A total of 62 adult male wild‐type (WT) C57BL/6 mice (22‐30 g; Charles River Laboratories) and 29 adult male LCN2 knockout (LCN2 KO) mice (a gift from Dr Xiaoli Chen, University of Minnesota, Breeding at University of Michigan Breeding Core) were used in this study.

### 2.2 Experimental groups

This study had two parts. In the first, WT mice underwent an endovascular perforation (SAH model) or a sham operation. All animals underwent an MRI after 4 hours and were then euthanized for either immunohistochemistry or Western blot to assess BBB leakage to albumin, LCN2 expression and the cell types expressing LCN2 and its receptor, 24p3R (also called SLC22A17). The second part compared BBB albumin leakage, oligodendrocyte loss and white matter injury 4 hours after SAH in WT or LCN2 KO mice using Western blot and immunohistochemistry.

### 2.3 Endovascular perforation model

Subarachnoid hemorrhage was induced by endovascular perforation technique as previously described.^9^ Briefly, mice were anesthetized with 5% isoflurane and core body temperature was kept at 37.5°C with a controlled heating pad. After induction of anesthesia, isoflurane was maintained at 1.5%‐2.0%. A middle skin incision was made to expose the common carotid artery, external carotid artery, and internal carotid artery. Following sectioning of the left external carotid artery, a 5‐0 monofilament suture was advanced into the left internal carotid artery until resistance was felt and carefully pushed further to perforate the artery. The suture was then withdrawn producing the SAH. Sham control mice underwent the same procedure without perforation.

### 2.4 MRI scanning

MRI was performed at 4 hours after SAH using a 9.4‐T Varian MR scanner (Varian Inc) with acquisition of T2 fast spin echo using a field of view of 20 × 20 mm, matrix of 256 × 256 mm, and 25 coronal slices (0.5 mm thick). Mice were anesthetized with 1.5% isoflurane throughout MRI examination. All image analysis was performed using ImageJ software.

### 2.5 SAH grade

The extent of SAH was assessed using a modified grading system as previously described.^9^ The basal brain, including brain stem, was divided into six segments. Each segment was assigned a grade from 0 to 3, depending on the amount of blood. The minimum SAH grade is 0 and maximum grade is 18.

### 2.6 Immunohistochemistry

Immunohistochemistry was performed using the avidin‐biotin complex technique. Sections were incubated in 1:10 goat or horse serum (Vector Laboratories) for 30 minutes and incubated overnight at 4°C with primary antibody. Polyclonal rabbit anti‐LCN2 antibody (1:100; Abcam), polyclonal goat anti‐mouse albumin antibody (1:1000; Bethyl Laboratories), polyclonal rabbit antimyelin basic protein (degraded MBP) antibody (1:300, Millipore), and monoclonal mouse anti‐GST‐π antibody (1:100; BD Biosciences) were used. Goat anti‐rabbit IgG (1:500; Bio‐Rad) and horse anti‐mouse IgG (1:500; Bio‐Rad) were used as the secondary antibody. Negative control procedures included omission of the primary antibody.

### 2.7 Immunofluorescent double labeling

For immunofluorescent double labeling, the following primary antibodies were used: polyclonal rabbit anti‐LCN2 (1:100; Abcam), monoclonal mouse anti‐LCN2(1:100; R&D systems), polyclonal rabbit anti‐SLC22A17 (24p3R; 1:50; Abcam), polyclonal goat antiglial fibrillary acidic protein (GFAP; 1:1000; Abcam), polyclonal goat anti‐ionized calcium‐binding adapter molecule 1 (Iba‐1; 1:400; Abcam), monoclonal mouse antiglutathione S‐transferase‐π (GST‐π;1:100; BD Biosciences), monoclonal mouse anti‐NG2 (1:200; Millipore), and polyclonal rabbit anti‐von Willebrand Factor (vWF, 1:200; Sigma‐Aldrich). The secondary antibodies were donkey anti‐rabbit IgG (H + L) Alexa Fluor 594 (1:500; Invitrogen), donkey anti‐goat IgG (H + L) Alexa Fluor 488 (1:500; Invitrogen), and donkey anti‐mouse IgG (H + L) Alexa Fluor 488 (1:500; Invitrogen). After sections were incubated with 10% normal donkey serum at room temperature for 30 minutes, sections were incubated overnight at 4°C with primary antibodies. After washing, sections were incubated with secondary antibodies for 2 hours. Double labeling was analyzed using a fluorescence microscope, and positive staining for GFAP, Iba‐1, GST‐π, NG2, and vWF was used to identify astrocytes, microglia, mature oligodendrocytes, oligodendrocyte precursor cells, and endothelial cells within white matter.

### 2.8 Cell counting

For quantification, 4 slices from each brain with each slide containing 4 fields from corpus callosum and external capsule were digitized using a microscope (×40 magnification). The number of LCN2 and GST‐π positive cells was determined. Degraded MBP immunoreactivity was also scored 0 to 3 (none‐extended) as described previously^10^ and summed over 6 fields. Image analysis was performed using ImageJ software.

### 2.9 Western blot analysis

Western blot analysis was performed as previously described.^11^ Briefly, white matter tissues were sonicated in western blot sample buffer. Protein concentration was determined by Bio‐Rad protein assay kit, and 30 µg protein samples were separated by sodium dodecyl sulfate‐polyacrylamide gel electrophoresis and transferred to a Hybond‐C pure nitrocellulose membrane (Amersham). Membranes were probed with primary antibody: polyclonal goat anti‐mouse albumin antibody (1:50 000; Bethyl laboratories). Antigen‐antibody complexes were visualized with the ECL technique. Image analysis was performed using Image J software.

### 2.10 Statistical analysis

Values are presented as the means ± SD. Statistical differences among groups were analyzed using chi‐square test, student's *t* test or one‐way ANOVA with Tukey‐Kramer post hoc test. Statistical significance was set at *P* < .05.

## 3 RESULTS

In this hyperacute study, mortality rates were 0% (0/40) and 10.5% (2/19) after endovascular perforation in WT and LCN2 KO mice, respectively. No sham mice died (n = 22 for WT; n = 10 for LCN2 KO).

### 3.1 White matter with T2 hyperintensity at 4 hours after SAH

**Figure 1:**
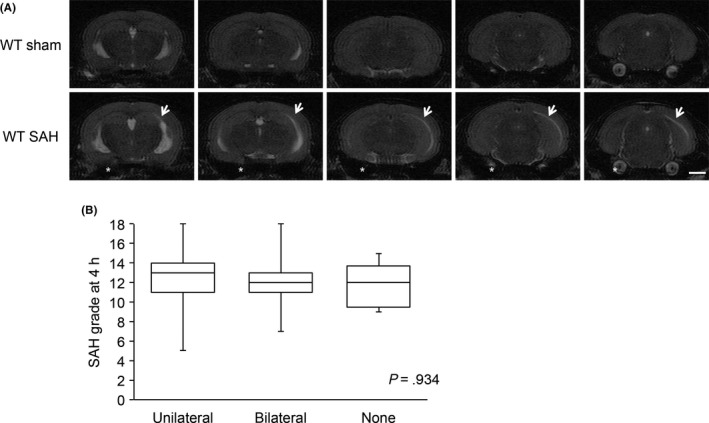
Appearance of T2 hyperintensity in white matter at 4 h after subarachnoid hemorrhage (SAH). A, Representative coronal T2‐weighted images (T2WI) of wild‐type (WT) mice at 4 h after SAH. Arrows indicate white matter, and asterisks indicate perforation side. B, Comparison of SAH grades in animals with unilateral (n = 20), bilateral (n = 15) or no (n = 5) T2 hyperintensities in white matter. Values are mean ± SD. There was no significant difference in SAH grade between the groups (ANOVA; *P* = .934). Scale bar = 1 mm

White matter T2 hyperintensities were observed in WT animals at 4 hours after endovascular perforation (Figure 1A). The overall incidence of T2 hyperintensities was 87.5%, occurring in 35 of 40 animals. Of the animals with T2 hyperintensities, 57% were unilateral (20/35: left side 2/20, right side 18/20), while 43% (15/35) were bilateral. The SAH grades were not different between animals with unilateral, bilateral and no white matter T2 hyperintensity (Figure 1B).

**Figure 2:**
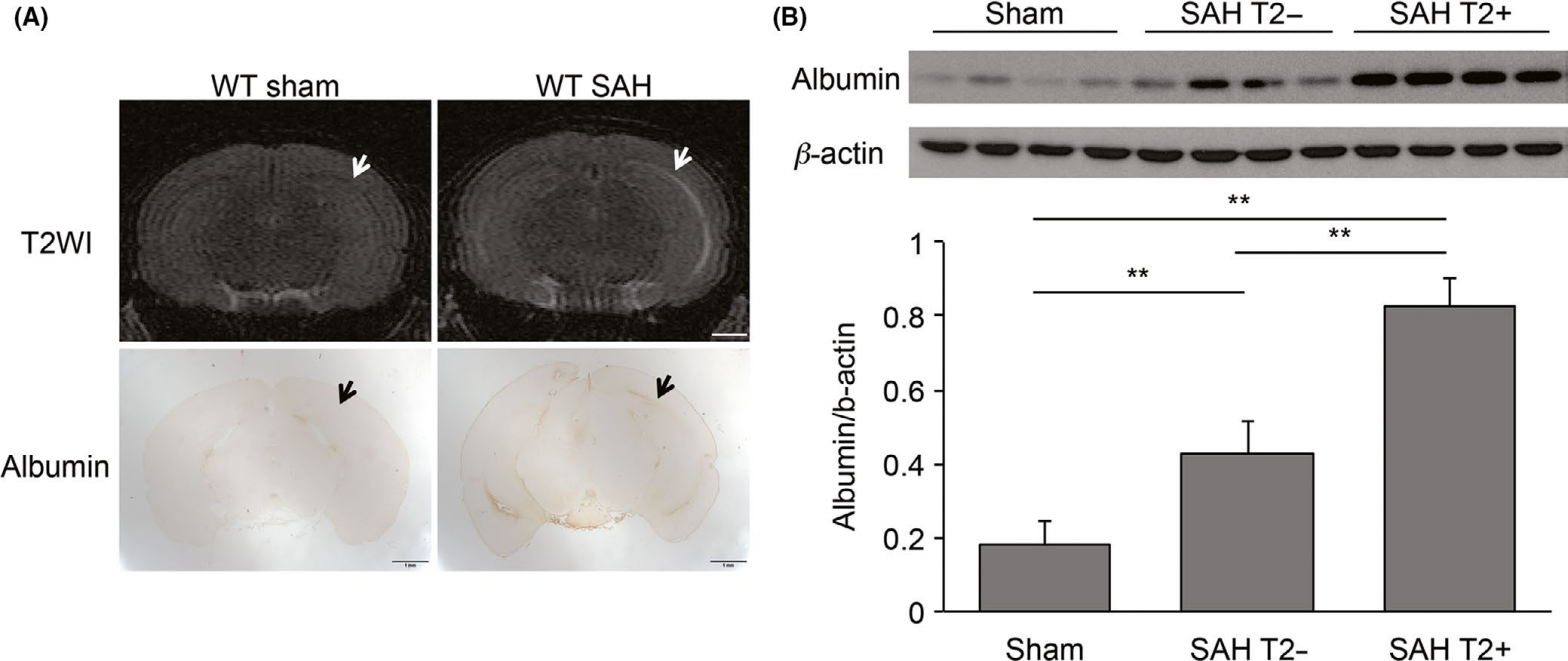
Subarachnoid hemorrhage (SAH) induced BBB disruption. A, Representative coronal T2‐weighted images (T2WI) and albumin immunohistochemistry of wild‐type (WT) mice at 4 h after SAH compared with a sham‐operated WT control. White and black arrows indicate white matter. Note the hyperintensity and area of albumin leakage in the SAH animal. Scale bar = 1 mm. B, Western blot of albumin levels in white matter from WT mice after a sham operation or an SAH. Samples were taken from SAH animals that had a white matter T2 hyperintensity on MRI (T2+) or did not (T2−). Albumin levels were quantified as a ratio to β‐actin (loading control). Values are mean ± SD; n = 4 for each; ^**^indicates *P* < .01

Subarachnoid hemorrhage also induced albumin leakage in the white matter at 4 hours. Spatially, the area of white matter albumin leakage was associated with T2 hyperintensity (Figure 2A). The level of white matter albumin leakage was significantly (*P* < .01) greater in animals with T2 hyperintensities (albumin ratio to β‐actin; 0.82 ± 0.07) than animals without T2 hyperintensities (0.42 ± 0.09) after SAH, although both were higher than animals undergoing a sham operation (0.18 ± 0.07, *P* < .01; Figure 2B).

### 3.2 LCN2 expression in white matter after SAH

**Figure 3:**
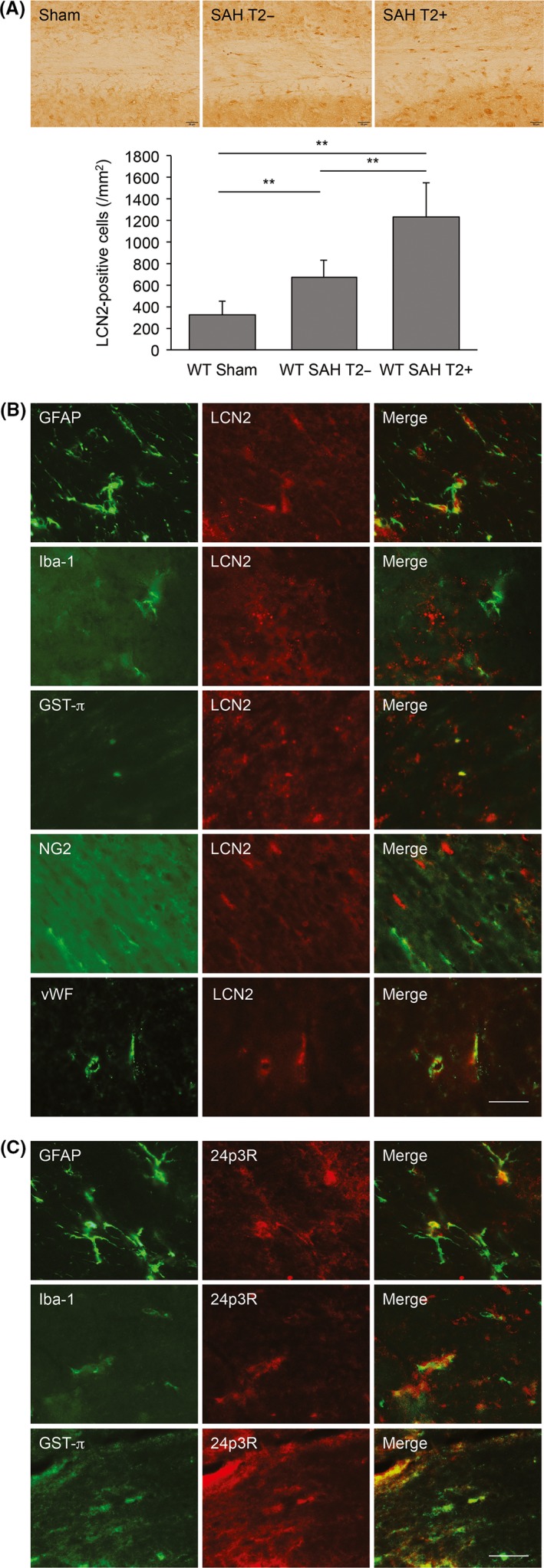
Subarachnoid hemorrhage (SAH) induced expression of lipocalin‐2 (LCN2) in white matter in WT mice. A, Examples of LCN2 immunoreactivity in white matter 4 h after a sham operation or after an SAH with (T2+) or without (T2−) a white matter T2 hyperintensity. Scale bar = 20 µm. The number of LCN2 positive cells in each group was quantified in the bar graph. Values are mean ± SD; n = 6 for each; ^**^ *P* < .01. B, Immunofluorescent double labeling of LCN2 with GFAP, Iba‐1, GST‐ π, NG2, and vWF in white matter at 4 h after SAH. LCN2‐positive cells colocalized with GFAP (astrocytes), GST‐ π (mature oligodendrocytes), and vWF (endothelial cells). Scale bar = 20 µm. C, Immunofluorescent double labeling of the LCN2 receptor, 24p3R, with GFAP, Iba‐1 and GST‐ π in white matter at 4 h after SAH. LCN2‐positive cells colocalized with GFAP (astrocytes), Iba‐1 (microglia), and GST‐ π (mature oligodendrocytes). Scale bar = 20 µm

Lipocalin 2 expression in WT mice at 4 hours after SAH was examined. The number of LCN2‐positive cells in white matter was significantly increased in animals with T2 hyperintensities after SAH (1229 ± 322 cells/mm^2^) compared with those without T2 hyperintensities (675 ± 157 cells/mm^2^; *P* < .01; Figure 3A). Both sets of mice had significantly more LCN2‐positive cells than animals undergoing a sham operation (323 ± 125 cells/mm^2^ *P* < .01).

Double labeling showed that LCN2‐positive cells colocalized with GFAP, GST‐π, and vWF positive cells, but not with Iba‐1 and NG2 positive cells (Figure 3B). Thus, LCN2 colocalized with astrocytes, endothelial cells, and oligodendrocytes but not microglia and oligodendrocyte precursor cells. Cells positive for the LCN2 receptor, 24p3R (SLC22A17), colocalized with GFAP, Iba‐1 and GST‐π immunoreactivity (Figure 3C), that is, astrocytes, microglia, and oligodendrocytes.

### 3.3 LCN2 deficiency results in less white matter BBB disruption after SAH

**Figure 4:**
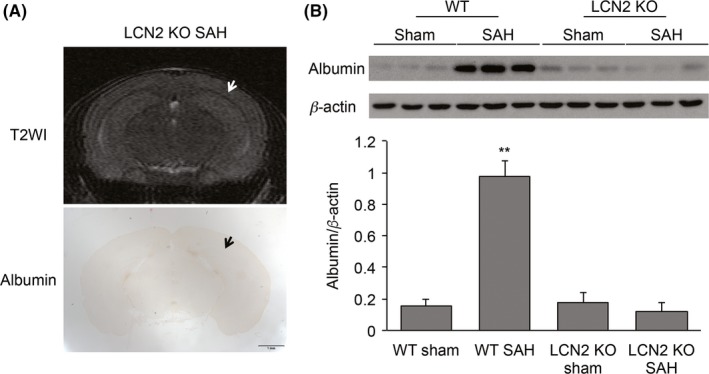
Lipocalin‐2 (LCN2) deletion prevented the occurrence of white matter T2 hyperintensities and blood‐brain barrier (BBB) disruption at 4 h after subarachnoid hemorrhage (SAH). A, Representative T2‐weighted MRI and albumin immunohistochemistry. White and black arrows indicate white matter. Note the absence of a hyperintensity area or increased BBB permeability. Scale bar = 1 mm. B, Western blot for albumin in white matter from wild‐type (WT) and LCN2 knockout (LCN2 KO) mice 4 h after an SAH or a sham operation. Albumin levels are quantified in the bar graph (ratio to β‐actin). Values are mean ± SD; n = 3 for each; ^**^ *P* < .01

To examine whether LCN2 deletion attenuates BBB disruption, WT and LCN2 KO mice were examined at 4 hours after SAH. All LCN2 KO mice had no white matter T2‐hyperintensity lesion after SAH (Figure 4A). The albumin leakage level in white matter in LCN2 KO mice with SAH was significantly less than in those in WT mice with SAH (albumin ratio to β‐actin; LCN2 KO mice; 0.12 ± 0.05, WT mice; 0.94 ± 0.10, *P* < .01; Figure 4B). We have previously found that there is no difference in SAH grade between LCN2 KO and WT mice (as assessed at 24 hours^4^). There was no difference in albumin leakage between WT and LCN2 KO mice undergoing a sham operation (Figure 4B).

### 3.4 LCN2 deficiency results in less oligodendrocyte loss and white matter injury after SAH

**Figure 5:**
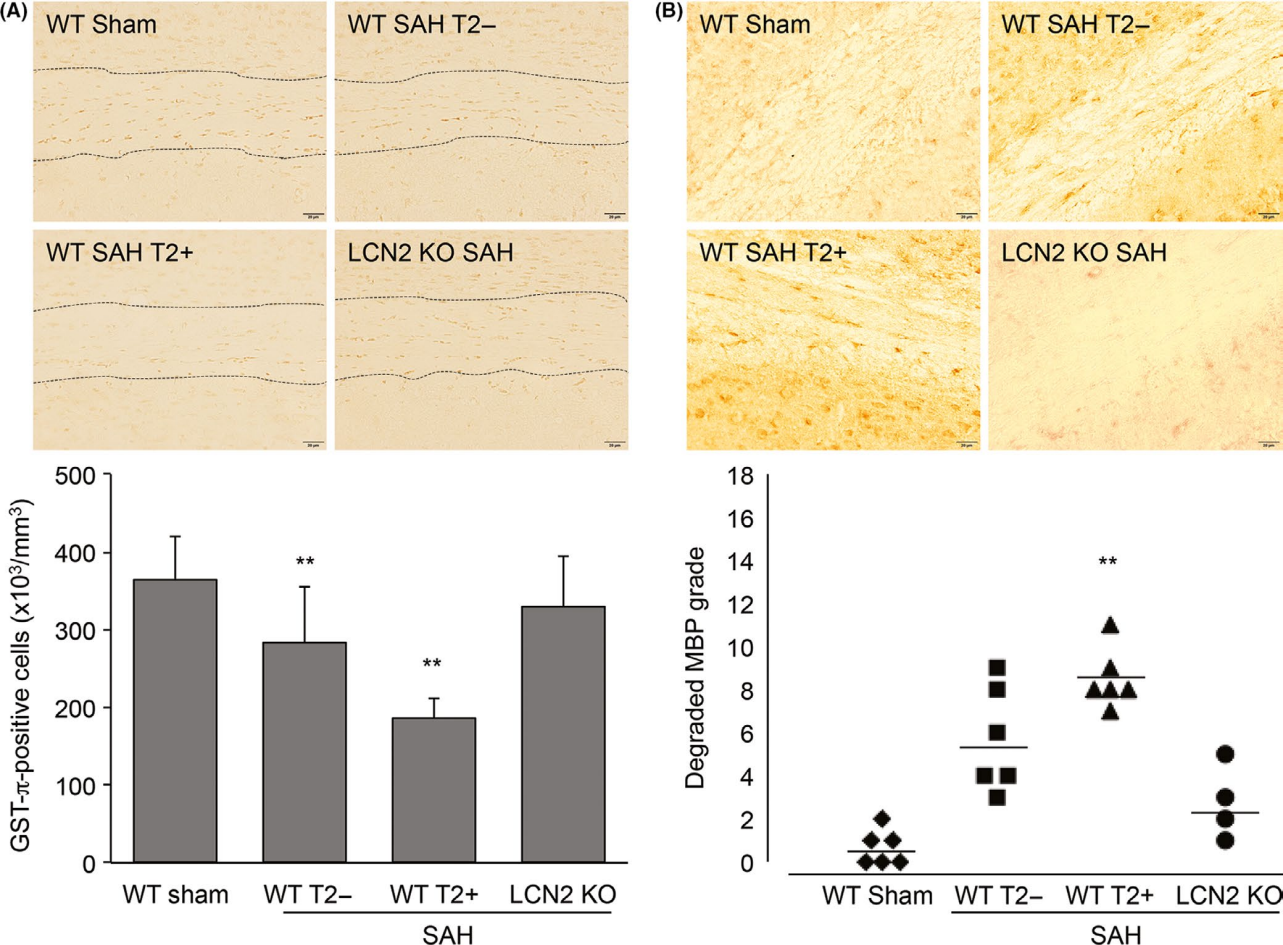
Lipocalin‐2 (LCN2) deletion attenuated oligodendrocyte loss and myelin damage at 4 h after subarachnoid hemorrhage (SAH). A, Representative GST‐π immunohistochemistry and quantification of GST‐ π immunoreactivity in white matter (between the black dotted lines) of wild‐type (WT) and LCN2 knockout (LCN2 KO) mice after SAH. Values are mean ± SD; n = 4 for each; ** *P* < .01 vs WT sham. B, Representative degraded MBP immunohistochemistry and quantification of degraded MBP immunoreactivity in white matter of WT and LCN2 KO mice at 4 h after SAH. Scale bar = 20 µm. Values are mean ± SD; n = 4 to 6, **P < .01 vs WT sham

To investigate the effects of LCN2 deficiency on oligodendrocyte loss after SAH, GST‐π immunohistochemistry (a marker of mature oligodendrocytes) was performed at 4 hours. Compared with sham‐operated WT mice (365 ± 55 cells/mm^3^), the number of GST‐ π positive cells in white matter was significantly (*P* < .01) reduced in animals with (185 ± 27 cells/mm^3^) and without (283 ± 71 cells/mm^3^) T2 hyperintensities (Figure 5A) although the loss of cells was greater in the former (*P* < .01). In contrast, in LCN2 KO mice with SAH, a decrease in the number of GST‐ π positive cells in white matter compared with sham‐operated animals was not observed (Figure 5A).

Immunohistochemistry for degraded MBP was also performed to evaluate the myelin damage (Figure 5B). MBP is a major component of the myelin sheath. WT sham‐operated mice had minimal evidence of degraded MBP. However, with SAH, there was increased degraded MBP, especially in animals with white matter T2 hyperintensity (*P* < .01). In contrast, in LCN2 KO mice with SAH, an increase in the expression of degraded MBP in white matter compared with sham operation was not observed (Figure 5B).

## 4 DISCUSSION

The major findings of the present study are (a) experimental SAH induced by endovascular perforation in mice resulted in very early (4 hour) T2 hyperintensities and BBB disruption in white matter; (b) SAH also resulted in increased LCN2 expression in white matter, with LCN2 being increased more in mice with T2 hyperintensities than those without; (c) the sources of LCN2 after SAH were astrocytes, endothelial cells, and oligodendrocytes, while the LCN2 receptor (24p3R) was found on astrocytes, microglia/macrophages, and oligodendrocytes 4 hours after SAH; (d) LCN2 deficiency attenuated SAH‐induced BBB disruption, oligodendrocyte loss, and MBP degradation in white matter.

In this study, SAH caused very early white matter T2 hyperintensities on MRI. Recently, we reported experimental SAH resulted in white matter injury, and almost all WT mice had T2‐hyperintensity area in white matter ipsi‐ and contralateral to the site of rupture at 24 hours after SAH.^4^, ^12^ However, in the present study, 57% of WT mice with SAH had unilateral white matter T2‐hyperintensity area at 4 hours after SAH. It is a report on MRI change in the hyperacute phase after SAH but also suggests there is preferential site of injury—contralateral to the site of vessel rupture.

Other studys suggest white matter is particularly vulnerable to injury by SAH, possible via multiple mechanisms including ischemia and trauma.^13^, ^14^ In experimental cerebral ischemia, T2‐hyperintensity lesions appear 24 hours after transient middle cerebral artery occlusion.^15^ In contrast, in experimental traumatic brain injury, T2‐hyperintensity lesions are already recognized at 6 hours after onset.^16^, ^17^ The present study revealed that most unilateral T2‐hyperintensity lesions were on the right side, the opposite side to the endovascular perforation. In addition, we have the data that T2 hyperintensity in white matter is found bilaterally at 24 hours after SAH.^4^ Hyperacute T2‐hyperintensity lesion of opposite side of perforation might be a contrecoup injury caused by physical compression from the subarachnoid blood. Then, a hypoxic state causes bilateral white matter injury in the brain. Alternately, there may be differential access of subarachnoid blood to the left and right lateral ventricles causing differential injury.

Brain edema and BBB disruption are important components of early brain injury, and brain edema is a risk factor for worse outcome after brain hemorrhage.^18^, ^19^ In addition, white matter injury leads cognitive dysfunction, depression, and motor deficits.^20^ Our previous studies showed that T2‐hyperintensity lesions coincided with albumin leakage.^8^, ^12^ However, those studies did not examine animals without T2 hyperintensities because almost all mice had extended T2‐hyperintensity lesions in white matter at 24 hours. Our current examined both animals with and without T2‐hyperintensity lesions in white matter. At 4 hours after SAH, even animals without T2 hyperintensities already had albumin leakage. This result suggests BBB disruption starts very soon after SAH onset before MRI changes and that those areas evolve into T2‐hyperintensity lesions.

Li et al^7^ recently examined BBB leakage and brain endothelial tight junction protein changes in a similar endovascular perforation model of SAH but in rat. They found evidence of a biphasic BBB disruption with an early opening as early as 30 minutes that peaks at 3 hours and a later disruption that peaks at 72 hours post‐SAH. Those results were mirrored by changes in the brain endothelial tight junction proteins, occludin and ZO‐1. Those findings and the results presented here both indicate that there is very early BBB disruption after SAH. Recent results in cerebral ischemia have also detected very early BBB disruption^6^ and therapeutically targeting the brain endothelium to reduce that disruption also ameliorated brain injury and neurological deficits.^6^, ^19^ The effects of targeting acute brain endothelial disruption after SAH on brain injury merits investigation.

We recently reported that LCN2 contributes to SAH‐induced BBB disruption at 24 hours.^8^ The present study provides evidence that LCN2 is rapidly (by 4 hours) upregulated in white matter after SAH. That upregulation was greater in animals with white matter T2 hyperintensities than those without, but even the latter had significant upregulation. Importantly, animals deficient in LCN2 had no early (4 hours) BBB disruption after SAH. This result indicates that LCN2 may be a biomarker of hyperacute white matter after SAH preceding the development of white matter T2 hyperintensities.

Our current results also demonstrated that LCN2 deletion attenuates the occurrence of T2 hyperintensities, BBB disruption, oligodendrocyte loss, and myelin degradation in the hyperacute (4 hours) phase after SAH. LCN2 deficiency has also been found to reduce BBB disruption and neuroinflammation and oxidative stress at 24 hours after ICH, thrombin induced hydrocephalus and SAH.^8^, ^21^, ^22^ The exact mechanisms by which LCN2 is involved in BBB disruption, oligodendrocyte injury, and myelin damage are still unclear. Some studies on ischemic stroke have proposed a hypoxia—HIF‐1α‐LCN2—vascular endothelial growth factor (VEGF)A axis signaling mechanism.^23^ The HIF‐1α‐LCN2 signaling pathway plays an important role in cancer and acute kidney injury.^24^, ^25^ SAH causes a hypoxic state in acute phase due to elevated ICP and decreased CBF.^2^ Numerous studies have reported that HIF‐1α is expressed in brain in rat models of SAH.^26^, ^27^, ^28^ VEGFA is a potent regulator of endothelial permeability and disruption of the hypoxia—HIF‐1 α‐LCN2—VEGFA axis may help preserve BBB permeability. Furthermore, the current study found that oligodendrocytes express the LCN2 receptor, 24p3R as well as LCN2 itself. LCN2 via its receptor may be involved in cellular iron uptake after hemorrhage potentially causing iron toxicity.^21^ Alternately, 24p3R was also expressed on microglial cells suggesting that LCN2 may have a role in SAH‐induced neuroinflammation. However, recent studies showed that endothelial cells also can express 24p3R^29^ and LCN2 may help to maintain BBB integrity in brain ischemia.^30^

## 5 CONCLUSION

In conclusion, SAH causes uni‐ or bilateral white matter T2 hyperintensities within 4 hours. That is associated with acute BBB disruption and oligodendrocyte death. In white matter with T2 hyperintensity, there is greater LCN2 expression and BBB disruption. Mice without LCN2 have reductions in BBB disruption, oligodendrocyte death, and axonal damage after SAH.

## CONFLICT OF INTEREST

We declare that we have no conflict of interest.
